# Identification of Novel Pro-Migratory, Cancer-Associated Genes Using Quantitative, Microscopy-Based Screening

**DOI:** 10.1371/journal.pone.0001457

**Published:** 2008-01-23

**Authors:** Suha Naffar-Abu-Amara, Tal Shay, Meirav Galun, Naomi Cohen, Steven J. Isakoff, Zvi Kam, Benjamin Geiger

**Affiliations:** 1 Department of Molecular Cell Biology, Weizmann Institute of Science, Rehovot, Israel; 2 Department of Physics of Complex Systems, Weizmann Institute of Science, Rehovot, Israel; 3 Department of Computer Science and Applied Mathematics, Weizmann Institute of Science, Rehovot, Israel; 4 Massachusetts General Hospital Cancer Center, Boston, Massachusetts, United States of America; 5 Department of Cell Biology, Harvard Medical School, Boston, Massachusetts, United States of America; University of Birmingham, United Kingdom

## Abstract

**Background:**

Cell migration is a highly complex process, regulated by multiple genes, signaling pathways and external stimuli. To discover genes or pharmacological agents that can modulate the migratory activity of cells, screening strategies that enable the monitoring of diverse migratory parameters in a large number of samples are necessary.

**Methodology:**

In the present study, we describe the development of a quantitative, high-throughput cell migration assay, based on a modified phagokinetic tracks (PKT) procedure, and apply it for identifying novel pro-migratory genes in a cancer-related gene library. In brief, cells are seeded on fibronectin-coated 96-well plates, covered with a monolayer of carboxylated latex beads. Motile cells clear the beads, located along their migratory paths, forming tracks that are visualized using an automated, transmitted-light screening microscope. The tracks are then segmented and characterized by multi-parametric, morphometric analysis, resolving a variety of morphological and kinetic features.

**Conclusions:**

In this screen we identified 4 novel genes derived from breast carcinoma related cDNA library, whose over-expression induces major alteration in the migration of the stationary MCF7 cells. This approach can serve for high throughput screening for novel ways to modulate cellular migration in pathological states such as tumor metastasis and invasion.

## Introduction

Cell migration plays a critical role in numerous physiological processes, including embryonic development, inflammatory responses, wound healing, and angiogenesis, as well as in pathological states such as tumor invasion and metastasis [1], [2]. To explore the mechanisms underlying the regulation of cell migration, a variety of qualitative and quantitative approaches have been developed. These include 2- and 3-dimensional time-lapse movies, tracking the migration of cultured or tissue-embedded cells [3], [4], wound-closure assays [5]–[7], matrix-permeation assays [8], [9] and “recording” of the cells' migration “history,” based on assays such as PKT formation [10]. The latter assay is widely used for studying the migratory activities of different cell types [3], [11], matrix remodeling [12], [13] and perturbation of cell migration by chemical or genetic modulators [14]–[19]. Such studies are of particular relevance to cancer cell motility, which is believed to reflect the invasive or metastatic potential of these cells in vivo [14], [20]–[23]. Thus, identification of chemicals that alter cell migration, or specific genes whose perturbation affects cell migration could potentially be used for the modulation of metastatic cell migration.

Our objective in the present study was to develop a PKT-based approach for tracking cell migration, which is reproducible, compatible with high-throughput microscopy, and provides quantitative information, morphological and dynamic, on the migratory process. We show here that while the PKT records the integrated history of migratory activity at a single time point, the quantitative imaging software, enables the calculation of both “static” parameters such as track length and area, and “dynamic” parameters such as migration rates, persistence, and lamellar activity.

The high-throughput migration assay described herein, and the imaging software developed for measuring different features of the migratory process, provide a rapid, reliable and quantitative approach for assessing cell migration in diverse cell types, cultured under varying conditions, and exposed to a variety of chemical or genetic perturbations.

## Results

### Development of a bead-based high-throughput PKT assay

Critical to the development of this PKT assay was the selection of suitable beads, with optimal dimensions and chemical properties (Table S1). The beads that were found most suitable for PKT assays applied to a wide variety of cell types were carboxylate-modified latex (CML) white polystyrene beads, with an average diameter of 340 nm, and a negative charge content of 184.7 µEq/g. These beads form a homogenous and visible monolayer; their attachment to the substrate is firm enough to prevent spontaneous detachment, but still susceptible to removal by migrating cells.

The surface chemistry of the beads was found to have a strong effect on the PKT assay: beads with an aldehyde-modified surface attached firmly to the substrate, and could not be removed by migrating cells. Beads with a sulfated surface tended to aggregate, yielding a non-uniform monolayer. Carboxylated beads, with or without additional sulfate groups, tended to form rather homogenous suspensions after centrifugation. The surface density of the carboxylate groups also affected track formation: a low charge density (23.9 µEq/g) caused the bead to interact strongly with the surface, such that many cell types failed to effectively remove the beads as they migrated. Beads with carboxylate groups of intermediate density (91.4 µEq/g) were found optimal for some adherent cells (e.g., H1299, REF52) but not for cells with weaker adhesions (e.g., MCF7; B16-F10). Beads containing carboxyl groups with a density of 160–185 µEq/g were found to be optimal for assays applied to a wide range of cell types.

Moreover, the diameter of the beads had a major effect on the visibility of the tracks and on the stability of the monolayer. Thus, small beads (<300 nm in diameter) could hardly be visualized, while large beads (∼1,000 nm in diameter) tended to detach from the surface and then spontaneously reattach, resulting in poorly defined tracks. The optimal bead diameter for automated PKT assays was found to be about 400 nm.

### Development of the automated microscopy system

PKT assays were recorded using a cell-screening microscope [24] equipped with a laser autofocus device [25]. The microscope operating program and the image acquisition software were written as an application within the UCSF PRIISM environment (http://msg.ucsf.edu/ive). For this application, images were taken using a 10×/0.4 objective, under transmitted light illumination. A light diffuser was inserted above the multi-well plate, to minimize non-homogenous illumination and avoid the shadows commonly caused by the narrow well walls.

The dedicated acquisition program controls the illumination; autofocusing and image acquisition steps for all selected fields within each well, and for all selected wells in the plate, while optimizing the experimental details (e.g. well number, field position, exposure time, objective, optical setting, and the like). In order to record a maximal number of complete cell tracks, images of adjacent fields were fused into a seamless montage, in which tracks spanning more than one image are merged at high precision (Figure S1).

### Quantification of migratory parameters, based on PKT morphometry

**Figure 1:**
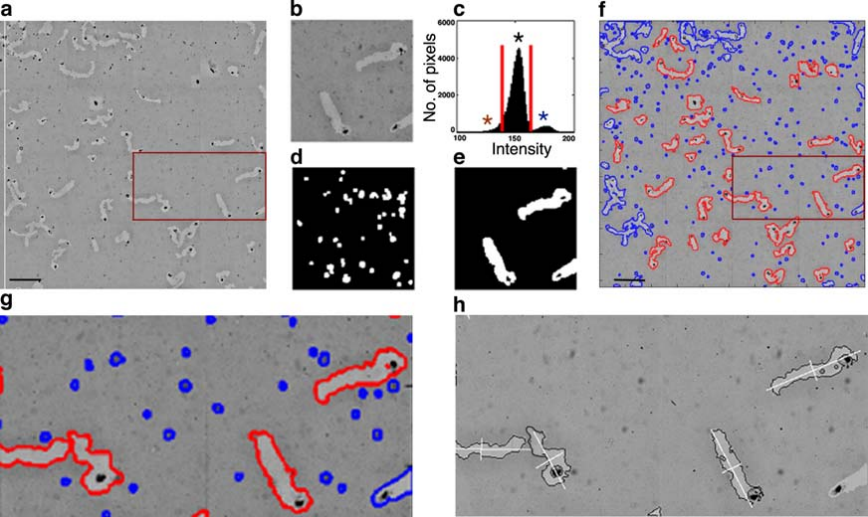
Computerized identification of individual PKT formed by H1299 cells. (a) A montage of 4×4 fields (1024×1024 pixels), each acquired using a 10×/0.4 objective. (b) A single field (512×512 pixels, binned 2×2) (c) Histogram of pixel intensity: the major peak (*) corresponds to background pixels; the minor peak of bright intensity (*) corresponds to track pixels; and the dark pixels represent cell bodies and debris (*). The red vertical lines mark the two thresholds separating the track pixels from the cells and the background. (d,e) Binary images, after applying thresholds. Pixels with intensities below the lower threshold (cells and debris) are colored white in (d), and pixels with intensities above the higher threshold (tracks) are colored white in (e). (f) All connected components of the entire montage are outlined: Tracks are outlined in red. Objects either too small or too large in area to be included in the image analyses, or located on the borders of the montage, are outlined in blue. (g) Enlargement of a segmented field following binary segmentation. (h) The same area depicted in (g), following multi-scale segmentation, and including the fine outline of the track and the track axes. Scale bars: 250 µm.

To identify individual tracks, images were subjected to “flattening”, thereby compensating for non-homogeneous illumination, smoothing and contrast enhancement. The resulting intensity histogram yielded a major peak, corresponding to the unperturbed background (Figure 1c, black asterisk); a high intensity peak corresponding to the bead-free tracks (Figure 1c, blue asterisk); and low-intensity pixels corresponding to bead-loaded cells (Figure 1c, red asterisk). By applying two binary thresholds, cells (Figure 1d), and tracks (Figure 1e) could be differentiated from each other. Debris, scratches, tracks with no, or more than one cell, intersecting tracks, and tracks extending beyond the border of the montage, were identified and discarded (Figure 1f, segments outlined in blue). To obtain fine definition of track borders, which were slightly blurred by the smoothing step (Figure 1g), track boundaries were recalculated from the original images, using multiscale segmentation analysis [26] (Figure 1h).

**Figure 2:**
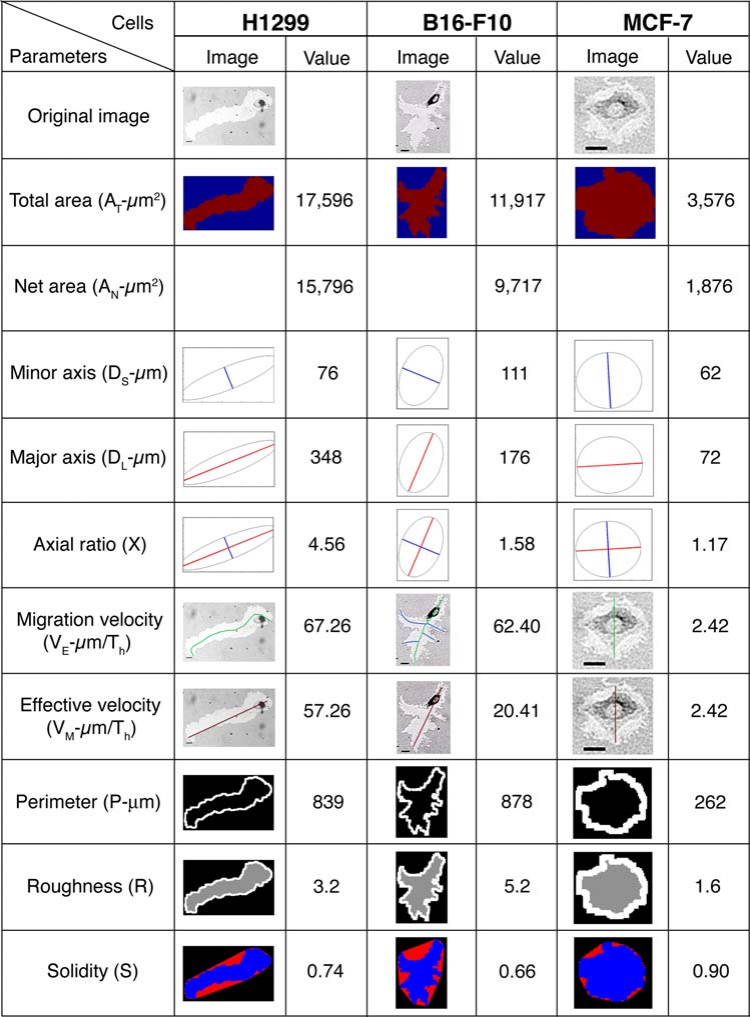
Morphometric parameters of PKT. The automatic calculation of the various morphometric parameters used in this study, are demonstrated using PKTs formed by H1299, B16-F10 and MCF7 cells. The calculated parameters include: Total track area (µm^2^), delineated in red; net track area after cell area is subtracted; minor and major axes (µm) of the best-fit ellipse; ratio of the axes; migration velocity [length of skeleton and branches (green+blue)/migration time], effective velocity [end-to-end distance (colored red)/migration time]; track perimeter; roughness [Perimeter^2^/(4π*Area)] and Solidity [track area (blue)/area of the convex hull (red+blue) enclosing the track]. The values indicated in the figure refer to the single track shown.

Following the segmentation step, we quantified the various morphometric parameters for each cell type. The track parameters that were automatically measured included track area, perimeter, major axis, minor axis, axial ratio and solidity. Track path length and end-to-end length were manually measured. The track parameters calculated from these morphometric parameters include persistence, effective velocity, average migration velocity, lamellar activity, and overall directionality. These morphological and “dynamic” parameters are defined in Table S2, and graphically presented in Figure 2 **.**

**Figure 3:**
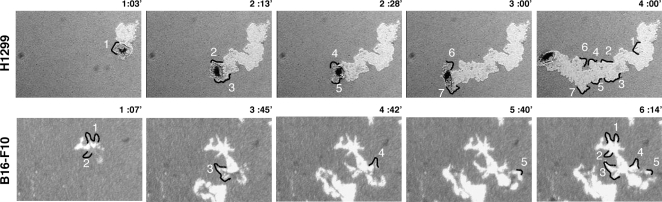
Track border roughness indicates lateral lamellar activity. Different time points are shown at which lateral lamellar activity of an H1299 (upper panel) or B16-F10 (lower panel) cells leave behind marks on the shape and roughness of the track border. (Full-length movies are available online as Movie S1 and Movie S2, respectively.)

Notably, even apparently similar parameters (e.g., “migration velocity” and “effective velocity”) can vary greatly. For example, B16-F10 cell, shown in Figure 2, exhibited almost the same migration velocity (62.4 µm/hr) as H1299 cell (67.26 µm/hr), while the latter cell type displayed a much higher effective velocity (57.26 µm/hr, compared to 20.4 µm/hr in B16-F10 cell), indicating a more persistent migration than the former. To assess “lateral” lamellar activity, which affects the width and roughness of track borders, we measured the track perimeter, and calculated roughness and solidity parameters. This analysis showed that while the perimeters of tracks formed by H1299 and B16-F10 cells were nearly the same, the roughness parameter was considerably higher in B16-F10 cell (5.2) than in H1299 cell (3.2), indicating higher lamellar activity in the melanoma cells. This finding was directly confirmed by time-lapse movies (Figure 3, and Movies S1 and S2).

### Application of PKT morphometry for measuring cell-type specific and drug-induced effects on migratory parameters

#### a) Different cell types produce PKT with distinct characteristics

**Figure 4:**
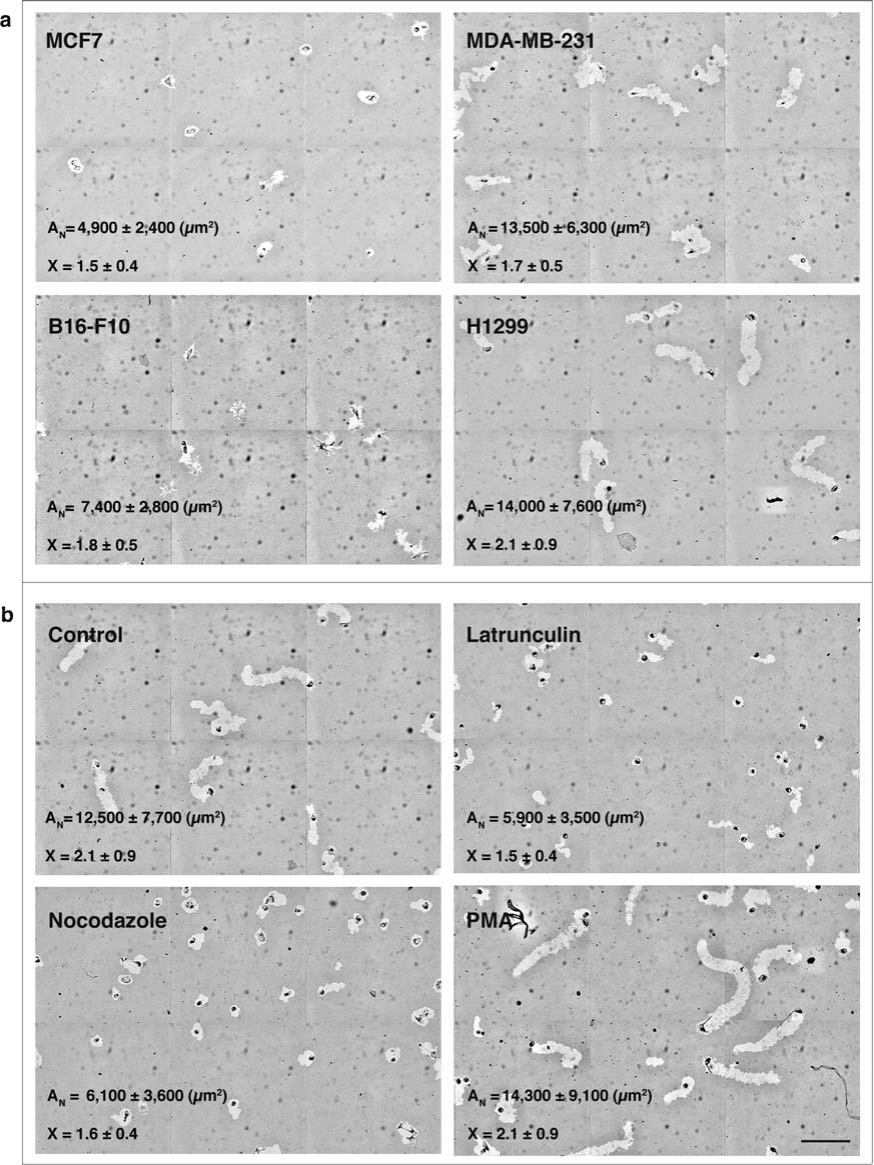
PKT features of different cell lines and the effects of cytoskeletal drugs on tracks parameters. (a) A montage of 2×3 images of the PKT of MCF7 cells, as well as for MDA-MB-231 cells, B16-F10 cells, and H1299 cells. Note the differences in track net area (A_N_) and axial ratio (X), depending on the cell line. (b) A montage of 2×3 image of H1299 control cells, which exhibit long migratory paths that are highly persistent. The montage images of the Latrunculin A (4 µM)- and Nocadazole (2.5 µM)-treated wells indicate inhibited cell motility; PMA (100 ng/ml)-treated well shows an increase in cell motility. Scale bars: 250 µm.

The PKT assay described herein was used to test the migratory behavior of a variety of cell lines. These include: B16-F10 and B16-F1 melanoma cells; MDA-MB-231 and MCF7 breast carcinoma cells, REF52, SV80 and NIH3T3 fibroblast lines; H1299 lung carcinoma cells, and several prostate carcinoma lines (DU145, PC3 and CL1). Four of these cell lines (MDA-MB-231, MCF7, H1299 and B16-F10) are utilized for the purposes of illustration (see Figure 4a and Table S3). MCF7 cells, for example, hardly migrate, producing only a small, bead-free zone around each cell, with an average net track area of 4,900±2,400 µm^2^ (n = 93). The B16-F10 melanoma cells produce branched tracks due to the extension of multiple filopodia and thin lamella largely perpendicular to the main migratory track, with an average net area of 7,400±2,800 µm^2^ (n = 124). The MDA-MB-231 cells are highly migratory metastatic cells, producing both long and wide tracks with an average net area of 13,500±6,300 µm^2^ (n = 149). H1299 cells are characterized by rapid and highly persistent migration, forming tracks with an average net area of 14,000±7,600 µm^2^ (n = 104).

#### b) The effects of cytoskeletal drugs on PKT structural and dynamic features

In order to determine whether our automated screening system was capable of detecting changes in specific migratory features induced by genetic or chemical perturbations, we treated H1299 cells with various compounds (e.g., Latrunculin-A, Nocodazole and PMA) known to affect cell motility. The effect of each drug on the different morphometric parameters was then measured (Figure 4b). Since the values for each PKT parameter did not appear to have normal statistical distributions, we based our comparison on changes in percentile values for each morphometric parameter, rather than on changes in average values (Table S4). Analysis of the PKT area of H1299 cells treated with the various inhibitors indicated that Latrunculin-A or Nocodazole markedly reduced track areas, axial ratios, migration velocities and roughness, compared to control cells. In the PMA-treated cells, the PKT area, major axis and calculated migration velocities increased (p<0.05), but the minor axis, axial ratios and solidity did not significantly differ from those of control cells.

### Application of the PKT assay to the identification of pro-migratory genes in a breast carcinoma-related gene library (BC1000)

The PKT assay described herein was used to screen a library of cancer-related genes for their ability to induce a migratory phenotype in largely stationary breast epithelial cells. For that purpose, we infected cultured MCF7 cells with retroviral vectors encoding 55 genes selected from the BC1000 library (Table S5) and tested their migratory activity by means of quantitative PKT screening.

**Figure 5:**
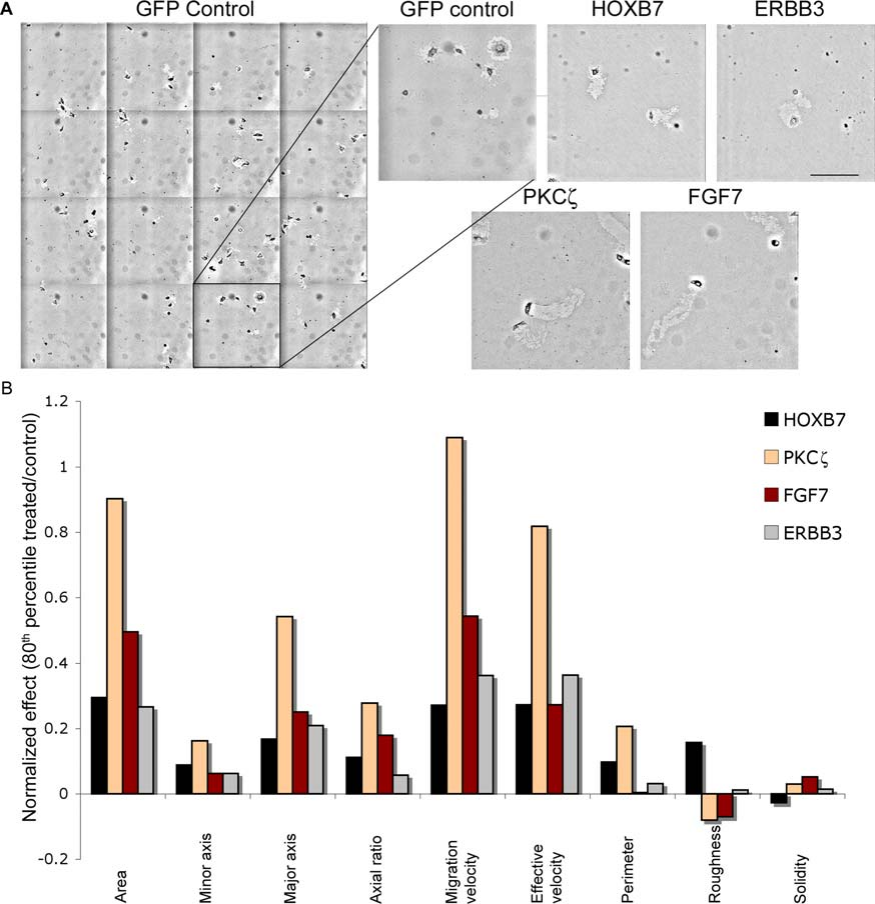
The pro-migratory effect of genes derived from the BC1000 library. (a) A montage of 4×4 images, comparing PKT produced by GFP-MCF7 control cells, to those produced by MCF7 cells expressing the BC1000-derived genes HOXB7, PKCζ, FGF7, and ERBB3. Magnification: 10×. Scale bars: 250 µm. (b) Calculated ratio between each of the migratory morphometric parameters of the different BC1000 library candidates, and those of control cells (GFP-MCF7). The primary statistical approach utilized was based on calculating, for each parameter, the “80^th^ percentile.” The normalized effect of GFP-control is always defined as zero: a zero value indicates no difference between the “80^th^ percentile” value of the candidate gene, and of the control cells. Numbers that are higher or lower than zero indicate an increase or decrease, respectively, in the 80^th^ percentile value of the tested parameters. (For additional details, see Table S6).

As shown in Figure 5a and Table S6, the PKT screen enabled us to identify four novel pro-migratory candidates: HOXB7, FGF7, ERBB3 and PKCζ. The 80^th^ percentile values, as well as the average and standard deviation, are presented in Table S6. While all four genes stimulated MCF7 cell migration, their effects on the various migratory parameters differed. Thus, while FGF7 and PKCζ clones induced the formation of long, persistent tracks, due to the enhancement of directional membrane protrusions, the elongated tracks, induced by HOXB7 and ERBB3 appeared to be associated with multiple membrane protrusions in all directions. Therefore, while prominence of directional “forward protrusions” cannot be directly assessed by track morphometry, it is apparent that enhancement of track length which is not accompanied by high roughness values is, most likely, driven by increased directional lamellipodial activity (Table S6 and Figure 5). It is interesting to note that in the screen of the BC1000 library, changes in migratory behavior were noted for another gene, namely MFGE8 (also known as breast epithelial antigen BA46). While the overexpression of this gene induced only minor enhancement in PKT area, it did greatly enhance track roughness, suggesting that it enhances non-directional membrane protrusion (Naffar Abu-Amara, unpublished data).

To determine the interrelationships between the various migratory parameters, we applied the Pearson correlation test to all PKT produced by unperturbed cells (Figure S2). This analysis revealed, for example, that track length (major axis and axial ratio) is negatively correlated with track solidity, indicating that the production of elongated tracks by the control cells is highly correlated with the lateral protrusive activity. This analysis was also applied to those cells expressing the different pro-migratory genes, in order to determine their capacity to disrupt the apparent interdependence between the various migratory parameters. These analyses demonstrate that, overexpression of PKCζ, HOXB7, and ERBB3 diminished the reciprocal relationship between track length and solidity.

Concerning the relationships between the axial ratio and the dimensions of the long and short axes, the control cells show the predominant contribution of the long axis to the axial ratio value, with the minor axis exerting a limited effect. In cells expressing FGF7 and PKCζ the axial ratio was mainly correlated to the major axis, while ERBB3 and HOXB7 affected the axial ratio by changing the track width. It thus appears that enhancement of overall cell migration by different pro-migratory genes can be achieved by selective modulation of a variety of dynamic cellular features such as cell polarization and protrusive membrane activity.

## Discussion

The primary objective of this study was the development of a quantitative assay for cell migration, which could measure multiple migratory features, and would be compatible with high-throughput screening. The major experimental challenge involved in designing such an assay is the apparent conflict between the rapid acquisition of vast amount of migration data, and the need to obtain “high content” information about the migratory behavior of many cells, including dynamic features such as migration velocity and lamellipodial activity. Since collection of direct dynamic information about cell migration is incompatible with high throughput, we therefore chose to explore indirect, yet reproducible and robust, approaches for obtaining such information. We propose herein that detailed morphometric analysis of PKT generated by individual migrating cells can provide such quantitative, morphological and apparently dynamic data.

The novel aspects of this study which deserve specific discussion are: (i) the choice of beads; (ii) track segmentation and morphometry; and (iii) statistical analysis of the data. The selection of beads for the PKT assay was based on three properties of the “migration field”: visibility of the track (primarily affected by the bead size, with an optimal bead diameter of 300–400 nm); their ability to form a homogenous layer (optimal with aldehyde or carboxylated surfaces; poor with sulfate-modified beads), and the capacity to be removed by migrating cells (optimal with carboxylated- and poor with aldehyde-modified beads). Notably, the susceptibility of carboxylated beads to removal by migrating cells is inversely correlated to the surface density of the carboxylate groups, enabling “fine tuning” of the bead layer for the particular cell type to be tested.

PKT segmentation and morphometry provided important insights into the basic features of the migratory process. These include the overall migratory activity of the cell, corresponding to the net PKT area; migration polarity, corresponding to the length of the major axis of migration (or the axial ratio); and “lateral” lamellar activity, measured by the track's solidity, among other values. Based on these measurements, several dynamic parameters were calculated, including average and effective migration rates, and lamellar activity. Naturally, the dynamic information, inferred from the static PKT morphology, is rather indirect, yet the high-resolution data [especially the roughness of track borders, measured by multiscale segmentation analysis [26]; (Figure 1h)] which could be correlated to dynamic information, based on time-lapse video microscopy (Figure 3). Once calculated for each track, the multiple parameters could be correlated to each other, either in control cells, or following chemical or genetic perturbation.

For our purposes, the PKT assay was utilized to discover novel pro-migratory genes in a breast carcinoma-related gene library. The rationale underlying this approach is that despite the obvious molecular complexity of the cell migration process, there might be “master genes” that could induce migratory features in a stationary cell. The unique capacity of the PKT assay we developed to highlight and quantify individual features of the migratory phenotype, could then link a given pro-migratory gene to specific migratory mechanisms. The new pro-migratory genes identified here include: HOXB7, ERBB3, PKCζ and FGF7. While all these genes are known to be “cancer-related”, the current study points to the possibility that their contribution to the malignant process might be related to their pro-migratory activity.

## Materials and Methods

### Preparation of bead-coated 96-well plates

Glass-bottomed 96-well plates (Whatmann, Inc., Clifton, NJ, USA, Cat. # 7706-2370) were incubated for 2 hr at room temperature, with 50 µl of 10 µg/ml fibronectin solution dissolved in PBS (Fibronectin, F-1141; Sigma Chemical Co., St Louis, MO, USA). The wells were then washed twice with PBS and coated with 340 nm white polystyrene latex beads (Interfacial Dynamics Corporation-Molecular Probes Microspheres Technologies, USA; Product no. 2-300; Batch no. 1344). The bead suspension (3.2 ml) was centrifuged for 5 minutes at 20,800×g, and the pellet resuspended in 4 ml PBS, until all visible bead clumps were dispersed. The sedimentation procedure was then repeated once more, after which the beads were resuspended in 7 ml PBS, to a final concentration of 0.9^12^ particles/ml. Aliquots of 70 µl of the bead suspension were added to each well, which had been pre-coated with fibronectin, and incubated at 37°C for 2 hr, followed by gentle washing with PBS (×5) using a plate washer (Colombus Plus, Tecan, Switzerland). Before cell plating, the PBS was replaced with 50 µl culture medium suitable for the particular cell type used in the assay (see Figure S3).

### Cell preparation for the PKT assay

MCF7 (ATCC-HTB-22), MDA-MB-231 (ATCC-HBT-26), and B16-F10 (ATCC-CRL-6475) cells were cultured in Dulbecco's Modified Eagle's Medium (DMEM); H1299 cells (ATCC-CRL-5803) were grown in RPMI-1640. Both culture media were supplemented with 10% FCS, 2 mM glutamine, 100 International Units/ml penicillin, and 100 µg/ml streptomycin (Biological Industries, Beit Haemek, Israel), and maintained in a 5% CO_2_ humidified incubator at 37°C. For the PKT assay, 200–400 cells (in 50 µl of medium) were cultured in each well. Depending on the typical track dimensions, as determined in preliminary experiments, the number of plated cells, and the time of incubation were calibrated to maximize the number of single, non-intersecting cell tracks. Typically, 200–400 cells/well and 7 hours of incubation were found to be optimal parameters for most cells.

### Pharmacological perturbations of PKT formation

To determine the effects of various pharmacological inhibitors on the H1299 cell line, cells were plated and incubated for one hour, after which they were treated with either 4 µM Latrunculin A; 2.5 µM Nocodazole; or 100 ng/ml PMA (Phorbol 12-mirystate 13-acetate). The cells were then incubated for an additional 4 hr, fixed with 3% paraformaldehyde and washed twice with PBS. Plates were either examined immediately by means of a screening autofocus microscope, or stored at 4°C for later inspection.

### Screening for migration-inducing genes

To screen for migration-inducing genes, we selected 55 candidate genes from the BC1000 library: (http://www.hip.harvard.edu/research/breast_cancer/index.htm), assembled at the Harvard Institute of Proteomics using literature-mining software [27]. This library consists of a collection of full-length cDNAs known to be associated with breast cancer development. The cDNAs are cloned into a puromycin- selectable retroviral vector (JP1520) [28] using the Creator™ recombination system (Clontech, Mountain View, CA). The 55 genes tested (see Table S5) were randomly selected from this library, and were generously provided by Prof. Joan Brugge (Department of Cell Biology, Harvard Medical School, USA). Genes were introduced into the MCF7 cells by means of retroviral infection. Each clone was tested for its impact on cell migration, using the PKT assay. As controls, we also generated JP1520 GFP-expressing MCF7 cells. The PKT assay was carried out in 96-well plates. Four hundred cells per well were seeded, and 8 wells were tested for each clone. The cells were incubated for 7 hours, and then fixed using 3% PFA. Data was collected using the autofocus-screening microscope.

### Statistical Analysis

Since the distribution of track parameters displays non-normal distributions, we used the percentile statistical tool to estimates parameters variability. For example, the 80^th^ percentile for track area is the value bellow which 80% of the tracks area are found.

Differences between control and treated cultures were evaluated for significance using the Two-Sample Kolmogorov-Smirnov goodness-of-fit hypothesis test. A p-value of <0.05 was considered to be statistically significant.

The Pearson's correlation test was conducted with values ranging from +1 (a perfect positive linear relationship between two tested variables) to −1 (a perfect negative linear relationship). A p-value of <0.0014 was considered statistically significant.

## Supporting Information

Table S1 Comparison of the properties of different beads used for the PKT assay.
(0.03 MB DOC)

Table S2 Parameters annotation.
(0.03 MB DOC)

Table S3 PKT analysis for the various cell lines.
(0.03 MB DOC)

Table S4 PKT analysis of H1299 cells treated by various drugs.
(0.03 MB DOC)

Table S5 List of tested genes.
(0.04 MB DOC)

Table S6 PKT analysis of MCF7 cells overexpressing a given pro-migratory gene.
(0.05 MB DOC)

Figure S1 A scheme describing the image acquisition and display process. (a) A template of a 96-well plate. (b) The positions of 52 fields that can be acquired within one well, using a 10× objective. (c) A montage of 4×4 images (1024×1024 pixels), corresponding to the marked area of the well. (d) A full-resolution image of one field (512×512 pixels) within the montage (marked in c). Scale bar: 250 µm.
(2.03 MB TIF)

Figure S2 Auto-correlation between the PKT morphometric parameters in control cells, and in cells expressing pro-migratory genes. Auto-correlation between the various morphometric parameters was calculated for the control (GFP-MCF7) library, as well as for cells overexpressing the different pro-migratory genes described in figure 5. Each rectangle is divided by a white line into two triangles; each triangle shows the correlation test of a different candidate. The p-value of each correlation result is indicated beneath the correlation score number.
(4.16 MB TIF)

Figure S3 Schematic outline of the 96-well plate preparation for the PKT assay.
(0.96 MB TIF)

Movie S1 PKT formation by H1299, plated on polystyrene beads. H1299 cells were plated on a glass-bottomed 35 mm dish covered with 10 µg/ml fibronectin, and coated with polystyrene beads. Cells were maintained at 370C in CO2-buffered RPMI 1640 medium with 10% FCS and penicillin/streptomycin antibiotics. Before transferring the cells to the microscope, the CO2- buffered RPMI 1640 medium was replaced with HEPES buffered pre-warmed medium. Images were acquired using a 10× objective, every 5 minutes over 5 hours.
(2.93 MB MOV)

Movie S2 PKT formation by B16-F10 melanoma cells, plated on polystyrene beads. B16-F10 cells were plated on a glass-bottomed 35 mm dish covered with 10 µg/ml fibronectin, and coated with polystyrene beads. Cells were maintained at 370C in CO2-buffered DMEM medium with 10% FCS and penicillin/streptomycin antibiotics. Before transferring the cells to the microscope, the CO2-buffered DMEM medium was replaced with pre-warmed HEPES-buffered medium. Images were acquired using a 10× objective, every 5 minutes for 7 hours.
(2.14 MB MOV)
