# Supplementary material for: Identification of Novel Pro-Migratory, Cancer-Associated Genes Using Quantitative, Microscopy-Based Screening
Source: PLoS One. 2008 Jan 23;3(1):e1457. doi: 10.1371/journal.pone.0001457 (PMC2195451; doi:10.1371/journal.pone.0001457)
Supplement: Table S1 — Comparison of the properties of different beads used for the PKT assay. (0.03 MB DOC) [file pone.0001457.s001.doc]

Supplementary Table 1: Comparison of the properties of different beads used for the PKT assay.

| Product#/ Batch # | Bead diameter  (nm) | Surface group and  charge content  µEq/g | | | Bead attachment strength | Monolayer quality | PKT  formation | Contrast |
| --- | --- | --- | --- | --- | --- | --- | --- | --- |
|  |  | Carboxyl | Sulfate | Aldehyde |  |  |  |  |
| 2-300/1867 | 310 | 23.9 | N/A | - | +++ | Homogeneous | - | N/R |
| 2-300/1041 | 320 | 201.2 | N/A | - | ++ | Homogeneous | **Clear | ++ |
| 2-300/1178 | 330 | - | N/A | 11.5 | +++ | Homogeneous | - | N/R |
| 2-300/2431 | 330 | 162.1 | 9.4 | - | ++ | Homogeneous | *Clear | +++ |
| 1-300/1053 | 340 | - | 3.6 | - | Non-homogeneous*** | | | |
| 2-300/1344 | 340 | 184.7 | N/A | - | ++ | Homogeneous | *Clear | +++ |
| 1-300/401 | 350 | - | 0.6 | - | Non-homogeneous*** | | | |
| 1-300/1955 | 350 | - | 2.1 | - | Non-homogeneous*** | | | |
| 1-400/1915 | 350 | - | 6.0 | - | Non-homogeneous*** | | | |
| 2-400/1049 | 400 | 91.4 | N/A | - | +++ | Homogeneous | **Clear | +++ |
| 2-1000/1685 | 1,000 | 446.0 | N/A | - | ± | Low bead density | Beads were floating close to the surface, and tracks were not produced by the cells | |

All the beads are negatively charged.

+ Low, ++ Medium, +++ High

* Suitable for a wide range of cell types.

** Suitable for specific cell lines only (H1299, REF52).

*** Beads tend to aggregate following centrifugation and resist further dispersion

N/A=Not Applicable, N/R=Not Relevant; PKTs were not formed.
