# Supplementary material for: Identification of Novel Pro-Migratory, Cancer-Associated Genes Using Quantitative, Microscopy-Based Screening
Source: PLoS One. 2008 Jan 23;3(1):e1457. doi: 10.1371/journal.pone.0001457 (PMC2195451; doi:10.1371/journal.pone.0001457)
Supplement: Table S2 — Parameters annotation. (0.03 MB DOC) [file pone.0001457.s002.doc]

Supplementary Table 2: Parameter annotation.

| **Parameter annotation**  **[dimensions]** | Explanation | **Measured/Calculated by** |
| --- | --- | --- |
| **A_T_ [µm^2^]** | PKT total area [cell area subtracted] | Automatically measured by Matlab: regionprops.area |
| **A_N_ [µm^2^]** | PKT net area [cell area subtracted] | Average cell area value was subtracted manually from the PKT area value |
| **P [µm]** | Track perimeter | Automatically measured by Matlab: regionprops.perimeter |
| **R** | Roughness, R= P^2^/(4π*A) | Manually calculated from A and P |
| **D_L_, D_S_ [µm]** | Major (long) and minor (short) axes of best fit ellipsoid, calculated from second moments about center of area | Automatically measured by Matlab: regionprops.MajorAxisLength/  matlab: regionprops.MinorAxisLength |
| **X** | Axial Ratio, X= D_L_ / D_S_ | Automatically calculated from D_L_, D_S_ |
| **A_CH_ [µm^2^]** | Convex hull area is the region between the smallest convex set that contains all the outside points of the track | Automatically measured by Matlab: regionprops.ConvexArea |
| **S** | Solidity = A/A_CH_ | Automatically calculated from A, ACH |
| **L [µm]** | Main track skeleton length | Manually defined; cell length values are subtracted |
| **B [µm]** | Sum of skeleton branches | Manually defined |
| **E [µm]** | End- to-end distance of track skeleton | Automatically calculated by the program |
| **T [h]** | Total migration time (hours) | Measured |
| **V_E_** | Effective velocity = E/T | Manually calculated from E and T |
| **V_M_** | Migration velocity = (L+B)/T | Manually calculated from L, B and T |
