# Supplementary material for: Identification of Novel Pro-Migratory, Cancer-Associated Genes Using Quantitative, Microscopy-Based Screening
Source: PLoS One. 2008 Jan 23;3(1):e1457. doi: 10.1371/journal.pone.0001457 (PMC2195451; doi:10.1371/journal.pone.0001457)
Supplement: Table S3 — PKT analysis for the various cell lines. (0.03 MB DOC) [file pone.0001457.s003.doc]

Supplementary Table 3: PKT analysis for the various cell lines.

| **Cells**  **Parameters** | **H1299**  N=104 | **B16-F10**  N=124 | **MCF7**  **N=93** | **MDA-MB-231**  **N=149** |
| --- | --- | --- | --- | --- |
| Net track area (µm^2^) | 14,000 ± 7,600 | 7,400 ± 2,800 | 4,900 ± 2,400 | 13,500 ± 6,300 |
| Minor axis (µm) | 106 ± 36 | 90 ± 19 | 75 ± 20 | 111 ± 30 |
| Major axis (µm) | 220 ± 97 | 150 ± 41 | 113 ± 29 | 188 ± 61 |
| **Axial ratio** | 2.1 ± 0.9 | 1.8 ± 0.5 | 1.5 ± 0.4 | 1.7 ± 0.5 |
| **Perimeter (µm)** | 710 ± 330 | 560 ± 161 | 400 ± 150 | 650 ± 260 |
| **Roughness** | 2.6 ± 1.0 | 3.3 ± 1.0 | 2.4 ± 0.8 | 2.4 ± 0.8 |
| **Solidity** | 0.8 ± 0.1 | 0.7 ± 0.08 | 0.8 ± 0.08 | 0.8 ± 0.08 |
| **Migration velocity (µm/h)** | 44 ± 17 | 40 ± 18 | 6 ± 3 | 58 ± 29 |
| **Effective velocity**  **(µm/h)** | 32 ± 13 | 17 ± 6 | 6 ± 3 | 26 ± 10 |
