# Supplementary material for: Identification of Novel Pro-Migratory, Cancer-Associated Genes Using Quantitative, Microscopy-Based Screening
Source: PLoS One. 2008 Jan 23;3(1):e1457. doi: 10.1371/journal.pone.0001457 (PMC2195451; doi:10.1371/journal.pone.0001457)
Supplement: Table S4 — PKT analysis of H1299 cells treated by various drugs. (0.03 MB DOC) [file pone.0001457.s004.doc]

Supplementary Table 4: PKT analysis of H1299 cells treated by various drugs.

| **Treatments**  **Parameters** | Control **n = 421 tracks** | **Latrunculin A**  **n = 602 tracks** | **Nocodazole**  **n = 588 tracks** | **PMA**  **n = 380 tracks** |
| --- | --- | --- | --- | --- |
| Net track area (µm^2^) | 12,500 ± 7,700  (18,200) | 5,900 ± 3,500  (8,200)  p=1.2x10^-43^ | 6,100 ± 3,600  (8,300)  p=4.6x10^-42^ | 14,300 ± 9,100  (21,000)  p=0.0221 |
| **Minor axis (µm)** | 100 ± 33  (125) | 79±19  (90)  p=2.0x10^-25^ | 78 ± 18  (91)  p=3.1x10^-22^ | 108 ± 42  (139)  N.S. |
| Major axis (µm) | 200 ± 98  (292) | 121 ± 44  (152)  p=4.1x10^-43^ | 123 ± 43  (155)  p=4x10^-38^ | 220 ± 104  (311)  p=0.047 |
| **Axial ratio** | 2.1 ± 0.9  (2.7) | 1.5 ± 0.4  (1.8)  p=1.3x10^-20^ | 1.6 ± 0.4  (1.9)  7.15x10^-17^ | 2.1 ± 0.9  (2.8)  N.S. |
| **Perimeter (µm)** | 615 ± 290  (865) | 380 ± 150  (455)  p=3.8x10^-43^ | 385 ± 140  (485)  p=3.0x10^-37^ | 695 ± 350  (980)  p=0.0124 |
| **Roughness** | 3.0 ± 1.1  (3.4) | 2.0 ± 0.7  (2.4)  p=4.0x10^-18^ | 2.1 ± 0.9  (2.5)  p=2.3x10^-14^ | 2.9 ± 1.4  (3.9)  p=0.01 |
| **Solidity** | 0.8 ± 0.1  (0.91) | 0.9 ± 0.07  (0.93)  p=3.54x10^-26^ | 0.9 ± 0.07  (0.93)  p=7.6x10^-21^ | 0.8 ± 0.1  (0.90)  N.S. |
| **Migration velocity**  **(µm/h)** | 42 ± 17  (53) | 10 ± 6  (15)  1.8x10^-17^ | 17 ± 11  (23)  4.6x10^-10^ | 59 ± 26  (84)  p=0.005 |
| **Effective velocity (µm/h)** | 36 ± 14  (47) | 8 ± 5  (11)  p=3.14x10^-18^ | 14 ± 8  (20)  p=3.5x10^-10^ | 46 ± 21  (67)  p=0.047 |

*N.S.=Not Significant

Average values and standard deviation

Values in parentheses = 80^th^ percentile values
