# Supplementary material for: Identification of Novel Pro-Migratory, Cancer-Associated Genes Using Quantitative, Microscopy-Based Screening
Source: PLoS One. 2008 Jan 23;3(1):e1457. doi: 10.1371/journal.pone.0001457 (PMC2195451; doi:10.1371/journal.pone.0001457)
Supplement: Table S5 — List of tested genes. (0.04 MB DOC) [file pone.0001457.s005.doc]

Supplementary Table 5: List of tested genes.

| **#** | **GI number** | **Gene symbol** | **#** | **GI number** | **Gene symbol** | **#** | **GI**  **number** | **Gene symbol** |
| --- | --- | --- | --- | --- | --- | --- | --- | --- |
| **1** | 12803738 | ERBB3 | **20** | 21707886 | BIRCS | **39** | 4757945 | CD83 |
| **2** | 15079546 | G1P3 | **21** | 184833 | IGF-1 | **40** | 6005730 | CHP |
| **3** | 14602762 | MDM2 | **22** | 18088238 | BMP4 | **41** | 4758077 | CSK |
| **4** | 15679935 | ENG | **23** | 13325245 | BIRC5 | **42** | 4504612 | CYR61 |
| **5** | 13177647 | MFGE8 | **24** | 13905041 | TPD52L2 | **43** | 11038657 | EDG4 |
| **6** | 14250476 | IL1B | **25** | 1628549 | EGFR | **44** | 15147344 | FGF7 |
| **7** | 14250621 | EMK1 | **26** | 4504158 | GRP | **45** | 5031702 | G3BP |
| **8** | 16307254 | SCYA2 | **27** | 15079240 | IGFBP5 | **46** | 10834983 | IL6 |
| **9** | 12653770 | CLCDN4 | **28** | 4507170 | SPARC | **47** | 13259537 | KAI1 |
| **10** | 12803364 | BC-2 | **29** | 15930064 | ABS | **48** | 21359833 | PPAP2B |
| **11** | 12653114 | GRN | **30** | 12653134 | FADD | **49** | 4505590 | PRDX1 |
| **12** | 17390233 | STRIN | **31** | 15341773 | LIMK2 | **50** | 4506274 | PTK9 |
| **13** | 15126675 | CFL1 | **32** | 15489286 | SCYB6 | **51** | 18656935 | PTN |
| **14** | 13177717 | DDIT3 | **33** | 14165485 | SPHK1 | **52** | 4507266 | STC2 |
| **15** | 14165514 | PRKCZ | **34** | 13623610 | STK15 | **53** | 4827037 | TPD52 |
| **16** | 12804382 | BFAR | **35** | 10947110 | ARG2 | **54** | 4885654 | WNT1 |
| **17** | 15929846 | HOXB7 | **36** | 20149550 | ARHGDIA | **55** | 20149551 | BCS1L |
| **18** | 13279010 | RAC1 | **37** | 4757771 | ARHI |  |  |  |
| **19** | 15278147 | TRIP10 | **38** | 10938017 | CCNB2 |  |  |  |
