# Supplementary material for: Identification of Novel Pro-Migratory, Cancer-Associated Genes Using Quantitative, Microscopy-Based Screening
Source: PLoS One. 2008 Jan 23;3(1):e1457. doi: 10.1371/journal.pone.0001457 (PMC2195451; doi:10.1371/journal.pone.0001457)
Supplement: Table S6 — PKT analysis of MCF7 cells overexpressing a given pro-migratory gene. (0.05 MB DOC) [file pone.0001457.s006.doc]

Supplementary Table 6: PKT analysis of MCF7 cells overexpressing a given pro-migratory gene.

| **Gene name**  **Parametersers** | GFP  control  **n = 230** | **HOXB7**  **n = 293** | **PKCζ**  **n = 303** | **FGF7**  **n = 189** | **ERBB3**  **n = 261** |
| --- | --- | --- | --- | --- | --- |
| Net track area (µm^2^) | 4,300 ± 2,000  (5,700) | 5,600 ± 3,600  (7,300)  p=4.8x10^-5^ | 7,500 ± 4,400  (11,000)  p=3.4x10^-20^ | 5,800 ± 4,000  (8,500)  p=5.2x10^-4^ | 5,700 ± 2,900  (7,200)  p=2.1 x 10^-17^ |
| **Minor axis (µm)** | 70 ± 20  (80) | 75 ± 20  (87)  p=0.004 | 80 ± 20  (93)  p=3.9x10^-6^ | 72 ± 20  (85)  N.S. | 75 ± 15  (85)  p=0.04 |
| Major axis (µm) | 100 ± 30  (120) | 120 ± 40  (140)  p=5x10^-5^ | 145 ± 55  185)  p=4.4x10^-20^ | 125 ± 60  (150)  p=4x10^-4^ | 120 ± 35  (145)  p=4x10^-9^ |
| **Axial ratio** | 1.5 ± 0.5  (1.8) | 1.6 ± 0.5  (2.0)  p=0.006 | 1.8 ± 0.6  (2.3)  p=3.8x10^-6^ | 1.7 ± 0.7  (2.1)  p=0.04 | 1.7 ± 0.6  (1.9)  p=1x10^-4^ |
| **Perimeter (µm)** | 345 ± 105  (420) | 380 ± 110  (460)  p=0.002 | 410 ± 140  (500)  p=7x10^-7^ | 360 ± 160  (420)  N.S. | 370 ± 100  (430)  p=0.01 |
| **Roughness** | 2.3 ± 0.7  (2.3) | 2.3 ± 0.7  (2.7)  p=1.4x10^-9^ | 1.9 ± 0.5  (2.2)  p=3.4x10^-42^ | 1.9 ± 0.7  (2.2)  p=6x10^-36^ | 2.0 ± 0.6  (2.4)  p=8.7x10^-25^ |
| **Solidity** | 0.83 ± 0.1  (0.92) | 0.8 ± 0.1  (0.89)  N.S. | 0.9 ± 0.2  (0.95)  p=6x10^-8^ | 0.9 ± 0.1  (0.97)  p=9.7x10^-11^ | 0.85 ± 0.08  (0.93)  p=0.006 |
| **Migration velocity (µm/h)** | 7 ± 4  (11) | 11 ± 5  (14)  p=7.8x10^-5^ | 16 ± 10  (23)  p=8.2x10^-24^ | 12 ± 9  (17)  p=2x10^-5^ | 12 ± 11  (15)  p=4.6x10^-8^ |
| **Effective velocity (µm/h)** | 7 ± 4  (11) | 11 + 5  (14)  p=2x10^-4^ | 14 ± 7  (20)  p=8.1x10^-116^ | 11 ± 8  (14)  p=1.7x10^-4^ | 11 ± 8  (15)  p=1x10^-6^ |

*N.S.=Not Significant

Average values and standard deviation

Values in parentheses = 80^th^ percentile values
