# Supplementary figures and images for: Identification of Novel Pro-Migratory, Cancer-Associated Genes Using Quantitative, Microscopy-Based Screening
Source: PLoS One. 2008 Jan 23;3(1):e1457. doi: 10.1371/journal.pone.0001457 (PMC2195451; doi:10.1371/journal.pone.0001457)

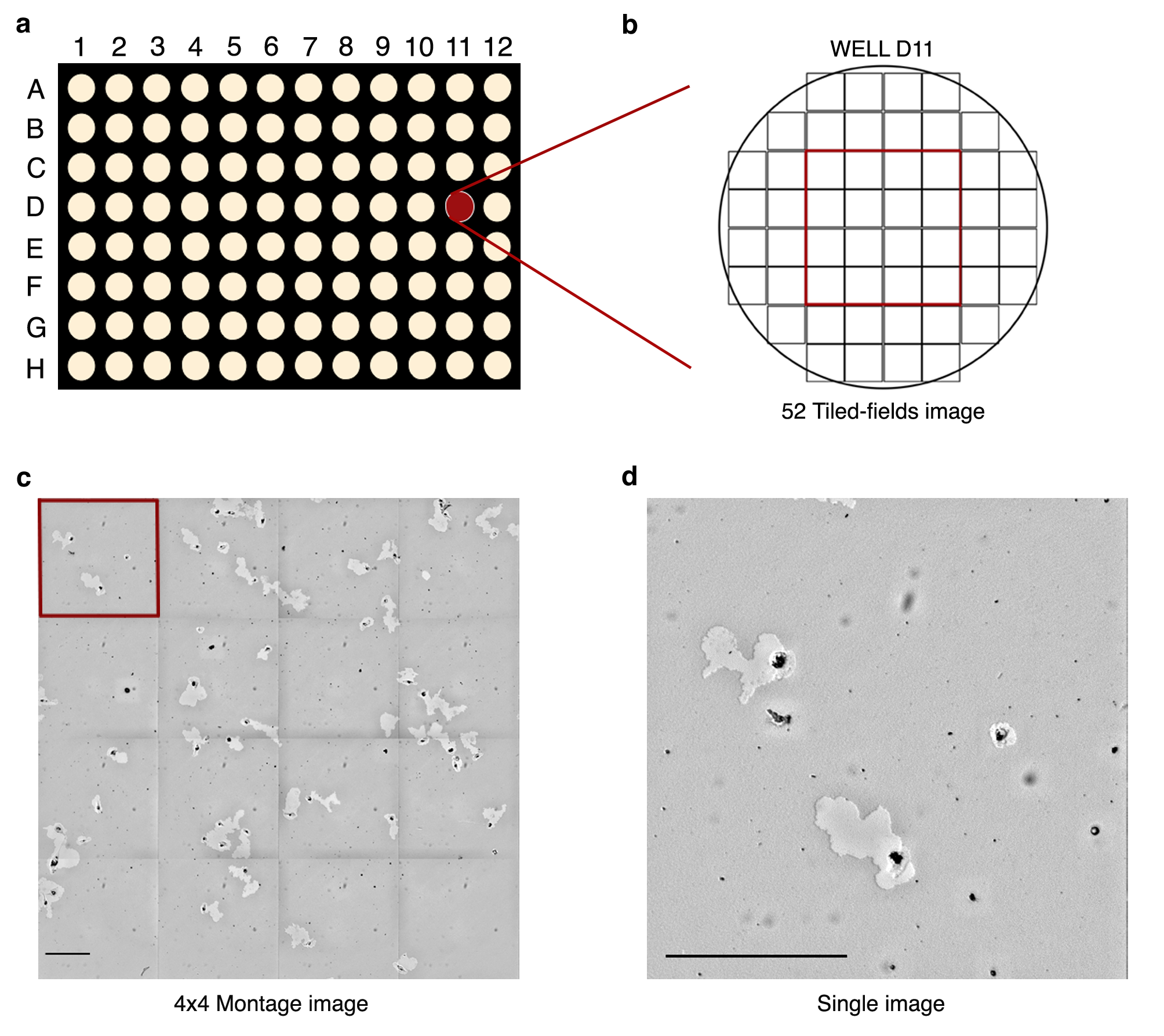

Supplement: Figure S1 — A scheme describing the image acquisition and display process. (a) A template of a 96-well plate. (b) The positions of 52 fields that can be acquired within one well, using a 10× objective. (c) A montage of 4×4 images (1024×1024 pixels), corresponding to the marked area of the well. (d) A full-resolution image of one field (512×512 pixels) within the montage (marked in c). Scale bar: 250 µm. (2.03 MB TIF) [file pone.0001457.s007.tif]

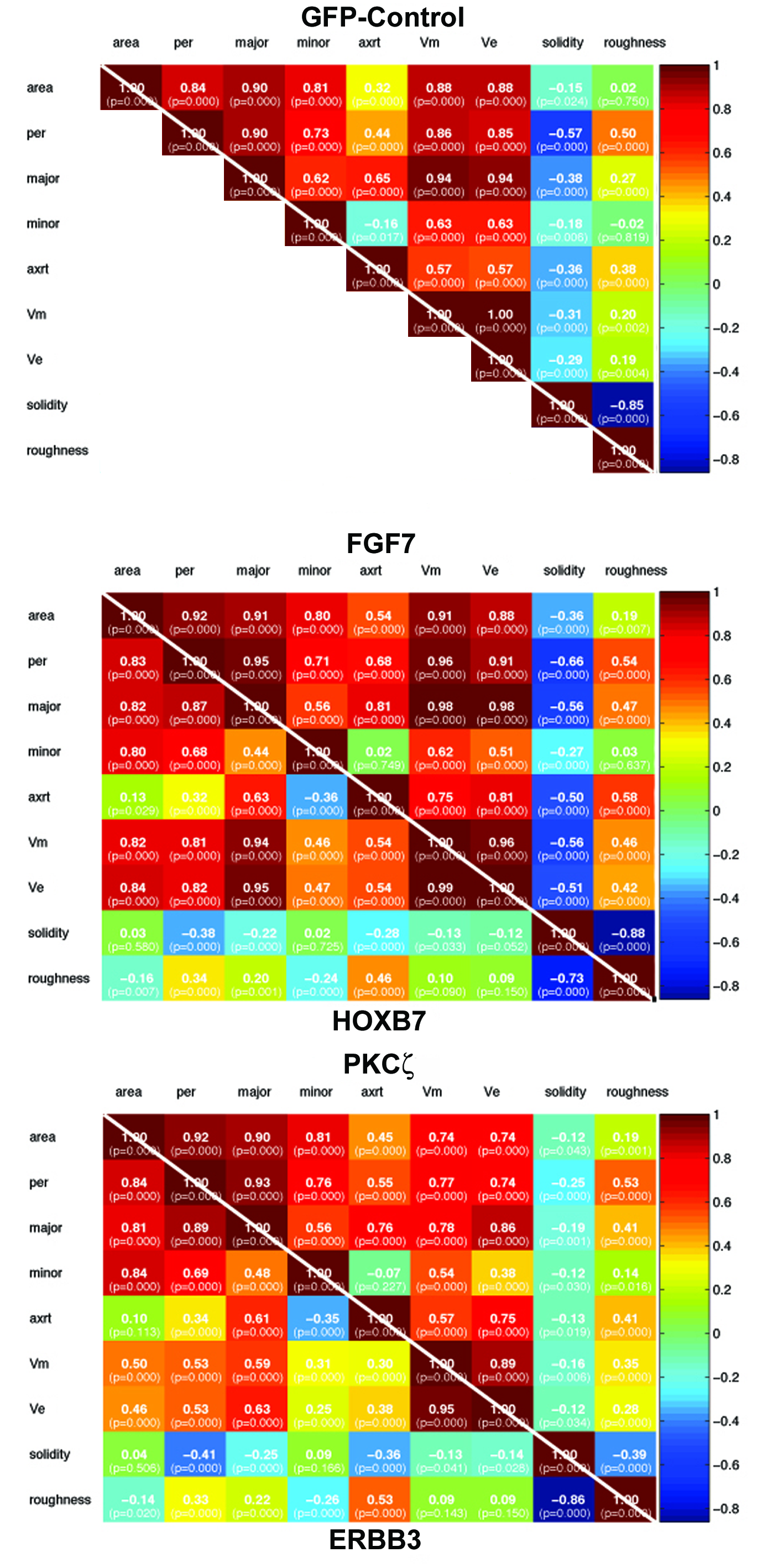

Supplement: Figure S2 — Auto-correlation between the PKT morphometric parameters in control cells, and in cells expressing pro-migratory genes. Auto-correlation between the various morphometric parameters was calculated for the control (GFP-MCF7) library, as well as for cells overexpressing the different pro-migratory genes described in figure 5. Each rectangle is divided by a white line into two triangles; each triangle shows the correlation test of a different candidate. The p-value of each correlation result is indicated beneath the correlation score number. (4.16 MB TIF) [file pone.0001457.s008.tif]

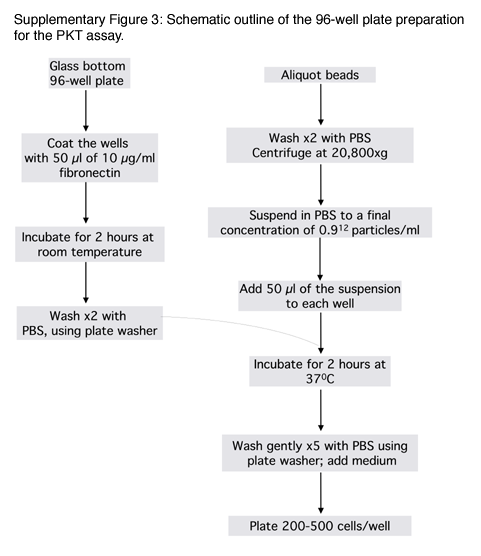

Supplement: Figure S3 — Schematic outline of the 96-well plate preparation for the PKT assay. (0.96 MB TIF) [file pone.0001457.s009.tif]
